# Responsible Governance for Mental Health Research in Low Resource Countries

**DOI:** 10.1371/journal.pmed.1001126

**Published:** 2011-11-22

**Authors:** M. Taghi Yasamy, Pallab K. Maulik, Mark Tomlinson, Crick Lund, Mark Van Ommeren, Shekhar Saxena

**Affiliations:** 1 Department of Mental Health and Substance Abuse, World Health Organization, Geneva, Switzerland; 2 Research and Development, The George Institute for Global Health, Hyderabad, India; 3 Department of Psychology, Stellenbosch University, Stellenbosch, South Africa; 4 Department of Psychology and Mental Health, University of Cape Town, Cape Town, South Africa

## Abstract

Blurb: Taghi Yasamy and colleagues identify challenges facing good research governance in low- and middle-income countries and provide suggestions for a way forward.

**Summary Points**

- Scaling up mental health services in low- and middle-income countries (LMICs) should be informed by a relevant evidence base to prevent harm and maximize effectiveness.
- International mental health research agenda prioritization exercises have highlighted priorities among which health system issues have gained more importance, and country-level adaptations of these priorities are needed.
- Mental health research governance mechanisms need to be improved at the national level in LMICs.
- It is essential to establish and institutionalize the general orientation of mental health research to deal with problems of organizational structure, research prioritization, insufficient involvement of local stakeholders and service users, relatively limited capacity and resources, and operational challenges.
- There is a need to balance expensive research with assessment of services and resources using low-cost methods, while building mechanisms to strengthen research capacity and to monitor the research process and outcomes.

## Introduction

Between 13% and 49% of the world's population develop neuropsychiatric disorders at some point in their life [1]. More and more evidence indicates that mental disorders and problems are common in all countries studied [2]–[4], and supports earlier projections that the burden of mental health problems is increasing in low- and middle-income countries (LMICs) as well [5]. Most people with these disabling conditions now live in LMICs, but at most one in five receives treatment and care [6]–[8]. In order to narrow this gap, the World Health Organization (WHO) launched the Mental Health GAP Action Programme (mhGAP) [9] with the objective of scaling up services for priority mental disorders using evidence-based interventions. In 2009, evidence profiles were compiled based on a systematic review of the literature for interventions that were to form part of the mhGAP Intervention Guide (mhGAP-IG) [10],[11].

These recent initiatives have once again shown that mental health research resources are sparse and unevenly distributed within LMICs, and that most research and publications originate from just 10% of this group of countries [12],[13]. Lack of good mental health research governance seems to be an important reason for the lack of mental health research from LMICs. In this article, we identify the challenges facing sound research governance in LMICs and provide suggestions regarding how research should be governed in this context, including suggestions for a way forward.

## Challenges Facing Good Governance

### Lack of an Organizational Structure for National Mental Health Research Governance

As is true for health research in general, the availability and strength of organizational structures that can lead and coordinate mental health research are limited and varied across LMICs. Most of the mental health research in LMICs is restricted to few larger countries (e.g., Argentina, Brazil, China, India, the Republic of Korea, and South Africa), and in three-quarters of mental health research, policy makers were not involved in planning or conducting the research [14]. The presence of mental health professionals in LMICs does not automatically translate to an effective “system” that governs mental health research. Universities in most LMICs do not have a strong link with the health system and psychiatrists do not receive much public health training [15]. Mental health research frequently does not follow health system needs [12].

### Confusion around the Priority Research Areas

Due to limited financial and human resources, allocation of assets for mental health research needs to be highly selective. Prioritization exercises in high-income countries do not necessarily apply to resource-poor countries. For example, a report from the United States National Institute of Mental Health in 2001 suggested basic science and developing new interventions were top priorities [16]. In contrast, however, priority-setting exercises in less affluent countries yielded different results. For example, *The Lancet*'s 2007 call for action on global mental health highlighted the need for research on health policy and systems and the scaling up and delivery of evidence-based treatments, while de-emphasizing research on the development of new interventions and technologies, drugs, vaccines, or medicines [17],[18]. Table 1 summarizes findings from global mental health agenda exercises, all of which prioritized health service research. Unfortunately local country-level adaptations of these research priorities were not undertaken as part of these exercises; but these are essential to make such recommendations locally relevant.

**Table 1:** Major global mental health research priority-setting exercises.

Priority-Setting Exercise	Method(s)	Scope/Regions	Major Results (Priorities)
*The Lancet* global mental health group, 2007 [17],[18]	Child Health and Nutrition Research Initiative (CHNRI)	Global	Identification of barriers in accessing health services; strategies to integrate needs into primary health care systems and ensure local delivery; health system research to ensure adequate provision, and where and how to deliver existing cost-effective interventions in a low-resource context.
Sharan et al., 2009 [12]	Literature search and mail survey	Africa, Asia, Latin America, the Caribbean	Epidemiology (burden and risk factors), health systems, and social science research. Depression/anxiety, substance use disorders, and psychoses; and children and adolescents, women, and people exposed to violence and trauma.
Khandelwal et al., 2010 [37]	Combined Approach Matrix (CAM)	Global	Awareness and advocacy, enhancement of research capacity, training for service delivery, and development of evidence-based policy.
Collins PY et al., 2011 [38]	Adapted Delphi method	Global	Integrate core service packages into primary health care, reduce cost and improve supply of medicines, provide effective and affordable community-based care and rehabilitation, improve children's access, and strengthen mental health component into training for all health care personnel.

National level priority-setting processes have been characterized as having a relative lack of genuine stakeholder engagement; a wide variation in terms of how priority-setting processes are documented; and an absence of a systematic appeal or feedback process for the identified priorities [19]. A recent exercise involving Peru, Uganda, and Nepal, for example, showed that involvement of grassroots workers can reduce the gap between research relevance and research excellence [20]. Despite the emphasis on health system research in global priority-setting exercises, health system or implementation research is rarely considered a priority. In these countries, biological research or research on new clinical interventions often outweighs research that focuses on delivering effective large-scale interventions within complex health systems that have possibly immediate public health impacts. Also, the issues of poverty and inequity are rarely addressed in research (see also [12]). Due to the fact that local funding for research is often absent, LMIC researchers often need to follow the research agendas of foreign donors rather than local needs.

### Research Capacity Constraints

Knowledge, attitude, and skills in the area of mental health research in LMICs needs to be improved [21],[22]. The number of psychiatrists, psychologists, and other behavioral scientists is low, and few have the time and capacity to plan, conduct, and disseminate highly competitive research [23]. In addition, involving local stakeholders in research by multilateral organizations is important [24]. A failure to do this can lead to acrimony affecting ownership of research results, inability to sustain long-term development of research policy, and failure to strengthen local research capacity.

There are other practical issues and context-dependent problems that hinder mental health research in LMICs. Low literacy, relatively poorer training in research methodology, inadequate research infrastructure, and a general apathy towards mental health conditions amongst the larger research community are a few examples. Lack of adequate funds and frequent population migration for better living conditions make large trials and cohort studies a challenge.

Some researchers are optimistic that people in LMICs generally have a reasonable understanding of research [25] and have been able to receive informed consent and conduct the research smoothly [26]. Yet, in many LMICs the capacity to prevent and manage research ethics violations is still limited and more needs to be done about research with people with serious mental health conditions in these countries. Problems with informed consent and the need for supported decision-making become more complicated among mental health service users even in higher income countries [27].

### Financial Constraints

Shortage of funds is a common constraint for mental health research in LMICs. Mental health research capacity is unequally distributed even within LMICs. Funding for health research is limited and a recent international survey showed that two-thirds of projects had received external funding [13]. The limited available funds in LMICs are often earmarked for communicable diseases and conditions named in the Millennium Development Goals and rarely available for mental health. Despite some advantages for the targeted areas, concern is growing over the impact of such vertical health programs on general health systems (see also [28],[29]). Funding for such projects is often in vertical silos, which tends to be detrimental to planning or developing other research and services. In addition, in the debates regarding funding for communicable versus non-communicable diseases, integrated approaches to research are lost [30]. The recent focus on mental health research available through some large international funding bodies has been predominantly on biological research—such as genomics—that is not a top priority for research in LMICs.

## Actions Required for Sound Mental Health Research Governance

### Institutional Arrangements

Governance of mental health research at the country level requires a mechanism for guidance and coordination. In cases where there is a center/unit for health research, mental health research should be established as a division or a branch of it. Such institutions need to set up formal institutional arrangements for engagement with experts in the area of mental health. Collaboration between health experts from different fields, including mental health, will lead to development of more effective programs that could have wider public health implications. For example, improving maternal mental health can influence nutrition status in young children [31]. Establishing collaborative research structures is also important to allow inclusion of key research stakeholders, such as academic institutions (with multi-disciplinary approaches), governmental and non-governmental organizations working in different sectors, and people with mental disorders themselves. Key to this is the development of a research culture and the stimulation of partnerships between researchers and policy makers. Researchers should be aware of the needs of the community and gaps in knowledge that prevent adequate policy development and conduct research that helps to answer those issues. Policy makers should also liaise with researchers and inform them about their needs while trying to understand the limitations of research.

This approach seems to be preferable to a situation where mental health research duplicates the mistake of other health research by being restricted within vertical programs. An integrated arrangement provides opportunities for piggy-backing mental health research on general public health research, which not only means more efficient utilization of a larger pool of funds, but also improved access to overall research funds for mental health. A welcome side effect of this approach would be to contribute to the de-stigmatization of mental health in general.

Finally, mental health research bodies need to develop appropriate stewardship, develop a long-term outlook and strategic plan, identify mental health research gaps and priorities, and monitor and coordinate relevant actions. Mechanisms need to be established to arrange for well-monitored international partnerships tailored to local needs. Positive examples of North–South collaboration with equality and efficiency have been reported [23]. Strengthening South–South partnerships, especially for neighboring countries, has also been suggested based on surveys that highlighted the advanced capacity of some middle-income countries [13],[32].

### Taking a Wide Range of Measures to fill the Information Gap

The information required for developing good policies and programs that lead to better mental health delivery models can come from alternative sources beyond traditional academic research proposals. In line with WHO's ongoing data collection exercise on mental health systems and resources, we discuss a logical flow of such information collection that contributes to the development of appropriate mental health services at different levels of care—macro, meso, or micro (see Table 2).

**Table 2:** Proposed knowledge collection from health system data collection to research.

	Scope	Global Outcome of the Project	Any Specific Outcome Related to a Country/Countries
**Step 1:** Project Atlas	Macro; global	Provides baseline data at a country level about mental health resources, policies, legislation	Information on resources is available for almost all countries, but does not include information on service gap
**Step 2:** WHO-AIMS	Macro; limited to selected LMICs	Provides more detailed information about mental health resources in selected LMICs and includes data about treatment practices and treated prevalence	Information is available for more than 60 countries thus far. Data on service gap is included.
**Step 3:** PRIME	Meso; Ethiopia, India, Nepal, South Africa, and Uganda	Provides data from research, based on mhGAP evidence-based interventions	To be assessed
**Step 4:** Small-scale research	Micro; research from individual settings	Data from smaller administrative units and communities helps in assessing the impact of the large programs in those communities and identifies problems and future needs that can help to improve them.	To be assessed

The WHO Mental Health Atlas [33]–[35] and the WHO Assessment Instrument for Mental Health Systems (WHO-AIMS) [23] are two instruments that provide information on mental health systems with very low cost. In 2001, Project Atlas highlighted the gaps in mental health resources across the world for the first time. This laid the stage for the next phase of more in-depth assessment carried out by WHO through the WHO-AIMS project. This study not only corroborated the findings that were obtained earlier through Project Atlas, but also the enormous treatment gap that existed in LMICs that provided such data. Projects such as the WHO Mental Health Atlas and WHO-AIMS are limited by being primarily based on government sources, but they can still provide some indicators to inform action and further research. They also have the scope of being repeated multiple times and thus contribute to the monitoring of progress in services development. Large epidemiological studies help to generate a sound evidence base, but these are expensive and may not give the best value for money in terms of monitoring progress on service delivery. It is in such situations that a stepwise pattern of data gathering carries importance.

A next step is to conduct evaluation studies of health system interventions that aim to scale up a core package of mental health services. Such an initiative has recently begun in the form of the PRogramme for Improving Mental health carE (PRIME) [36], a research consortium led from the University of Cape Town, with trial sites in Ethiopia, India, Nepal, South Africa, and Uganda. This consortium exemplifies the partnership between researchers and policy makers noted above. The final step is to conduct intervention studies (including trials) to evaluate the effectiveness and cost-effectiveness of specific interventions in local settings. It is important to emphasize that these steps are iterative—for example, ongoing local intervention studies can inform the development of policy and services, alongside macro level data collection.

Countries that apply and share such globally employed data collection and mapping instruments not only use the data to inform their national mental health policies and programs, but also contribute to global knowledge that enhances overall improvements in mental health at the global level.

As a complementary step, program evaluation should be added. Such evaluation should include measures of economic and social cost, as well as qualitative information to inform future projects.

### Tailoring Programmatic Solutions to Challenges

LMICs have similarities and differences in terms of their mental health research requirements [12],[13]. In Table 3 we have summarized a menu of options for sound governance of mental health research.

**Table 3:** Challenges and proposed arrangements for sound governance of mental health research.

Challenges	Steps to Overcome Challenges	Examples
Lack of structure or exclusion of mental health from health research governance mechanisms	• Establish a mental health research body within public health research institutions. Include mental health experts.	• In Ethiopia mental health specialists hold senior positions within university administrations, and this has contributed to higher quantity and quality of mental health research.
Research results are not useful	• Conduct a prioritization exercise with a participatory approach, involve users and key informants.• Use qualitative methods, involve consumers and key informants to assess needs.• Involve local stakeholders in multilateral research at all stages.• Monitor and evaluate research activities. This is critical to introduction of corrective measures and modifying the protocol as needed. Keep an eye on trends of research and publications.	• In the Mental Health and Poverty Project (MHaPP), Ministry of Health partners were involved in the development of the proposal and design of the studies, and participated in the interventions and publication of research findings in Ghana, South Africa, Uganda, and Zambia [39],[40].• Consultation with grassroots aid workers in Peru, Uganda, and Nepal influenced rating of research options [22].• A scientometric study in Iran identified preferences in mental health research that needed to be rectified [41].
Shortage of financial resources	• Plan and manage fundraising for sustainable “research for action” programs.• Be cost sensitive. Avoid costly epidemiological studies as a first option. Apply available data and reviews before embarking on fresh data generation. Where applicable, use secondary data from the country or similar contexts for planning.• Use low-cost options like WHO-ATLAS and WHO- AIMS to gather knowledge and assess the services.• Integrate mental health research into other public health research.	• To maximize available resources, a mental health screening tool has been introduced into the routine national Demographic and Health Survey in South Africa.• The National Health Survey of Iran initially did not have a mental health component. Based on advice from mental health experts, simple tools and semi-structured interviews were included in the survey and basic mental health data obtained provided useful information that was applied for both planning and advocacy [42].
Low capacity in terms of human resources	• Increase the profile of mental health in academic teaching and research training.• Foresee mechanisms for capacity building in all mental health research. All funded research should include a standard section on how capacity of local researchers will be increased, and what the expected outcomes will be.• Provide incentives for mental health research among mental health professionals [43]. Encourage mental health professionals to take up research as a career option.• Develop skills in areas of biostatistics, health economics, qualitative data analyses, and health policy and health services research.• Provide access to international literature.	• New programs are being developed to improve capacity for mental health research in LMICs, e.g., programs by TPO in Nepal, Sangath Centre in Goa, India, or Centre for Public Mental Health at University of Cape Town in South Africa.• In Iran, mental health research methodology workshops have been added since 1993 to the health system research methodology training workshops for medical science academics [44].
Research results are not applied	• Involve policy makers and mental health care providers in research, including the early design and proposal development stage.• Plan an effective dissemination strategy in advance to maximize the impact across different consumers. For example, plain lay language explanations would be needed for lay persons, while succinct policy briefs would be needed for policy makers highlighting the public health impact of the research.	• As part of the MHaPP, researchers conducted semi-structured interviews with a range of mental health stakeholders in four countries. The policies prioritized through this process were used to conduct interventions at macro, meso, and micro levels in collaboration with the Ministry of Heath and its partners [40].
Other issues (research ethics, consent, etc.)	• Develop skills and knowledge about research ethics and internationally accepted ethical guidelines• Develop good data management skills and incorporate steps to ensure data confidentiality• Low literacy may necessitate adaptations to methods to achieve meaningful consent.	• In 2009, an international group involved researchers from LMICs and identified key recommendations on ethical issues in conducting mental health and psychosocial research in humanitarian settings [45].• In Sri Lanka, researchers studied the capacity of individuals in understanding research as a requirement to receive informed consent [27].• In Pakistan, adaptations in the method such as naming “therapy” as “training” was helpful [46].

MHaPP, Mental Health and Poverty Project.

## Conclusion

There is a huge need and a growing demand for mental health services in LMICs. This requires a strong information base generated in the same countries. Locally conducted research would provide more direct evidence for interventions. But the service gap and the information gap go together. Low resource countries face a range of challenges that leads to little or inappropriate research. They need to use their limited financial and human resources for mental health research as effectively as possible. They need sound governance of their mental health research to achieve this, which requires the following:

- Organizing a structure for mental health research integrated within the available health research institutions;
- Developing a long-term outlook and strategic plan;
- Conducting a well-designed prioritization exercise. According to several international priority-setting exercises, mental health system research is the top priority;
- Raising awareness and developing a culture to understand and facilitate mental health research;
- Finding locally acceptable solutions for generating the required data such as application of qualitative methods and assessment of mental health systems by using alternative low-cost methods such as WHO-AIMS and the WHO Mental Health Atlas;
- Setting up routine information systems such as electronic medical systems, disease registries, and treatment outcome databases in LMICs with due consideration of confidentiality issues;
- Planning and managing fund raising, saving through integration within other health research, and rendering research efficient and sustainable, making the best use of available secondary data and research results from similar context;
- Capacity building for mental health research, inclusion of a capacity-building plan within any major research project, and information-sharing with policy makers and stakeholders on the benefits and potential utility of research;
- Establishing quality control, monitoring, and evaluation mechanisms for mental health research, observing ethical issues carefully, and following the trend of mental health research and publications;
- Planning dissemination from the start, involving policy makers in research governance to ensure knowledge translation; and
- Searching locally relevant innovative solutions for emerging challenges against mental health research.

This more strategic approach to research governance has the potential to strengthen the planning, execution, dissemination, and use of mental health research in LMICs.

## Abbreviations

LMIC: low- and middle-income country
mhGAP: Mental Health Gap Action Programme
mhGAP-IG: mhGAP Intervention Guide
PRIME: PRogramme for Improving Mental health carE
WHO: World Health Organization
WHO-AIMS: WHO Assessment Instrument for Mental Health Systems
